# Case Based Learning in Teaching Pharmacology to Undergraduate Medical Students

**DOI:** 10.7759/cureus.29187

**Published:** 2022-09-15

**Authors:** Preeti Garg, Sangeeta Bhanwra

**Affiliations:** 1 Pharmacology, Government Medical College & Hospital, Chandigarh, Chandigarh, IND

**Keywords:** learning, pharmacology, perceptions, students, case based learning

## Abstract

Background

With the new competency-based curriculum coming into effect we need to introduce newer teaching-learning methods to help students improve their learning and acquire necessary knowledge and skills. Hence to integrate pharmacology with clinical sciences and involve the students in their learning process, case-based learning (CBL) has been found to be very successful. Therefore the present study was planned to introduce CBL as a new teaching-learning method in pharmacology and to know the perceptions of the students regarding this method.

Objectives

The study aimed to introduce CBL to Bachelor of Medicine and Bachelor of Surgery (MBBS) students as a method of teaching pharmacology and the perceptions of the students regarding CBL were noted.

Methodology

After seeking institutional review board approval and sensitizing the students and faculty about this new teaching-learning method, CBL sessions were conducted on a few topics. Perceptions of the students were recorded on a feedback questionnaire and the results were analyzed.

Results

Ninety-five percent of students agreed that CBL helped in better comprehension of the concepts. Ninety-six percent of the students found CBL interesting and 96% of students said that CBL will help them correlate pharmacology clinically.

Conclusions

The students felt that CBL is an effective learning tool and would promote the clinical application of pharmacology.

## Introduction

Restructuring our way of teaching pharmacology is the need of the hour so as to determine how clinical pharmacology knowledge is applied in patient care, as documented by Vasundara et al. in a survey conducted by them [1]. Several new teaching and learning methods have been tried by various researchers, like the introduction of problem-solving interactive seminars or integration of two or more subjects, in an attempt to teach more effectively, and the results have come out in favor of newer methods [1-3].

Pharmacology involves a large multitude of concepts that cannot be learnt and retained by conventional teaching. In order to help learners enhance their overall academic performance, we need to focus on their progress in learning, retention, recall, and practical application [1]. This requires their active involvement. One of the simple approaches to facilitating learner participation is case-based learning (CBL) [2]. CBL is employed in every country on Earth to cover a portion of the curriculum or the entire curriculum. In the business environment, the technique is frequently employed for learning. According to reports, the approach emphasizes active learning and is student-centered. The adoption of several related CBL strategies is advantageous to the students. At the beginning of their careers, they gain better self-directed learning skills, communication skills, and decision-making abilities. The ultimate goal of medical students is to be competent physicians, and for this, they need to have a strong foundation of knowledge as well as skills. It has been demonstrated that knowledge of basic sciences gradually fades during clinical practice unless its relevance and utility in clinical situations are emphasized during the formative years of learning [3].

CBL is an educational paradigm based on andragogy that integrates multiple disciplines. It is an opportunity for the student to assimilate knowledge constructively. CBL is quite instrumental in honing soft skills and encourages critical thinking along with lifelong learning [2,4]. Cases are designed to be similar to real-life scenarios. They provide background information about a patient as well as supporting data such as clinical signs, symptoms, and laboratory tests. Based on this, the students work as a team, using their analytical and problem-solving skills, to devise a management strategy for the given case. The faculty members act as moderators, leading the discussion in the right direction and making sure it stays on track.

Many studies have shown that CBL is an effective method for teaching pharmacology, so the current study was designed to introduce CBL for teaching pharmacology to second-professional (Phase II) Bachelor of Medicine and Bachelor of Surgery (MBBS) students and collect their perspectives on this new teaching method in our setting. Hence, this study aims to introduce case-based learning (CBL) as a teaching and learning method for pharmacology and to assess the perceptions of students after introducing CBL in pharmacology.

## Materials and methods

The questionnaire-based research was carried out in the department of pharmacology after getting approval by the Institutional Ethics Committee of Adesh Medical College & Hospital, Shahbad, with certificate number AMCH/BIO/2018/11/016. The sample size was calculated by having a confidence interval of 95% and the population of the area and the individuals enrolled. Among 150 second-year professional students, 135 students who attended all the sessions on CBL were included in the study and those who didn't attend all the sessions prior were excluded. The faculty and students were sensitized regarding CBL. The participants were also given written material regarding CBL. Case scenarios on four topics (hypertension, anemia, peptic ulcer, and malaria) were developed with the assistance of clinical experts and faculty members in the department. Before beginning the study, written informed consent was taken from the students. Each topic was covered in two sessions, one week apart. The students were given the topics of the cases to be taken up in the succeeding class along with the reference resources and learning materials for self-study two days in advance. The students were divided into two batches of 75 each (Batch A and B) for practical classes. For the conduct of CBL, Batch A and B were further subdivided into 7 subgroups with 10-11 students in each subgroup under the supervision of one faculty member each. Eight sessions of two hours each were conducted for each batch. In the first session, the predecided topic in the form of clinical case scenarios was given to each subgroup in Batch A. The students were encouraged to interact and deliberate with each other to find answers to the given case.

In the subsequent session, the students refined and discussed their answers. One student from each subgroup then presented their case to the whole batch, and the facilitator cleared their doubts further. A feedback questionnaire that was pre-validated by members of the Institute's medical education unit was given to the students to gather their perceptions about this new teaching method (Table 1). The same procedure was followed for batch B and for the remaining topics.

**Table 1:** Feedback questionnaire to know the students’ perceptions of case-based learning as a teaching-learning method in pharmacology CBL: case-based learning; SDL: self-directed learning

S. No.	Questions
1.	CBL helps in better understanding of the concepts of pharmacology
2.	CBL stimulated my interest in pharmacology
3.	CBL will help me correlate pharmacology with clinical sciences
4.	CBL enhances motivation toward SDL (Self-directed learning)
5.	The cases facilitated active discussion
6.	The discussion sessions improved interaction between faculty and students.
7.	CBL helped me develop my communication and attitudinal skills
8.	CBL is a useful preparation in clinical problem solving
9.	Other topics in pharmacology should also be taught by CBL
10.	Future batches should also be taught by CBL
11.	The course was well organised
12.	I enjoyed case-based learning
13.	What are the advantages of CBL in your opinion?
14.	What are the limitations of CBL?

Students who attended all of the sessions were given feedback forms to complete at the conclusion. The questionnaire consisted of 12 questions based on a 5-point Likert scale (strongly disagree, disagree, neutral, agree, strongly agree) and two open-ended questions. The data was then quantitatively analyzed using Microsoft office excel and the results were expressed as percentages.

## Results

Ninety-five percent (95%) of the students were of the opinion that CBL helps in a better understanding of the concepts of pharmacology. The majority of the students said that CBL stimulated their interest in pharmacology (Table 2).

**Table 2:** Perceptions of the students

Questions	Strongly agree ( n %)	Agree ( n %)	Neutral (n %)	Disagree ( n %)	Strongly disagree ( n %)
Better understanding of concepts	37	58	4	1	0
Stimulated my interest	33	41	22	4	0
Helped correlate pharmacology with clinical sciences	51	45	4	0	0
Enhanced self-directed learning	30	56	9	4	1
Facilitated active discussion	37	41	15	6	1
Improved interaction between faculty and students.	41	33	19	6	1

Ninety-six percent (96%) of students were of the view that CBL would help them correlate pharmacology with clinical sciences, CBL enhances SDL (self-directed learning) was also agreed upon by more than 86% of the students. Seventy-four percent (74%) of students agreed that CBL enhances interaction between faculty and students and improves their soft skills, especially communication skills. About 89% of students were of the view that a few other important and difficult topics in pharmacology should also be taught by CBL. Almost all the students said that CBL should be used as a TLM ( teaching-learning method) for future batches. The majority of the students gave an opinion that all the sessions were well organized and they enjoyed case-based learning (Table 3).

**Table 3:** Perceptions of the students

Questions	Strongly agree ( n %)	Agree ( n %)	Neutral ( n %)	Disagree ( n %)	Strongly disagree ( n %)
Developed my soft skills	45	44	11	0	0
Useful for preparation in clinical problem solving	37	33	30	0	0
Other topics should also be taught by CBL	51	38	7	4	0
Future batches should also be taught by CBL	44	45	11	0	0
Course was well organised	56	33	11	0	0
I enjoyed CBL	37	44	15	4	0

In Table 2 there are students' responses to open-ended questions in the feedback form, as well as some suggestions such as teaching-learning methods should be taught more frequently and should be a regular component of the MBBS curriculum; learning through CBL will help us in clinics where we can apply this knowledge; and these sessions should be conducted to cover important and difficult topics like autonomic nervous system (ANS) and central nervous system (CNS), which bring in more clarity of concepts. The students said that CBL would help in building interpersonal relationships.

## Discussion

The educational system has undergone a sea of change from traditional learning methods being substituted by self-directed learning and active participation of the students [5]. CBL is a student-centered teaching method that acts as a stimulus and creates self-directed learning environments for the students to explore more and enhance their performance and analytical skills. CBL is a structured approach to dealing with clinical case scenarios. The major stakeholders are the students, whose opinions matter the most [6-7].

In our study, 95% of the students were of the opinion that CBL brought clarity of concepts and would help them correlate pharmacology with clinical sciences. CBL will help students apply the knowledge they have gained to real-life situations. Similar findings have been observed in a few other studies too [8-11]. The majority of the students were of the view that CBL motivates them toward self-directed learning. They feel encouraged to seek out additional resources. The same has been demonstrated by Gupta et al. in their study [12].

CBL is quite instrumental in enhancing their communication skills. They interact with each other, actively participate, discuss, fill in the gaps in their learning, and work as a team. Various studies have concluded that CBL improves students' self-reported analytical and communication abilities, as well as their confidence, contentment, enthusiasm, and involvement [13-15]. Since it is difficult to retain pharmacology lessons, the students wanted that some important and difficult topics in pharmacology, namely ANS, CNS, and cardiovascular system (CVS), should also be taught by CBL.

As Kamat et al. reported in their study, 89% of students gave a favorable response to the introduction of CBL to the subsequent batches [16]. Similar findings have been reported by several other studies where CBL has been studied and found to be having a good impact on the learning of the students [15,17].

CBL can be used to spark a keen interest in the subject and a desire to learn more about it, along with the acquisition of the required knowledge, skills, and attitude. It also encourages self-evaluation and critical reflection. This approach, like any other teaching-learning method, is not impeccable. This strategy necessitates much planning and effort at multidisciplinary levels, coordination, and organization in advance. Also, it is not an exam preparation activity, so as time goes by, students may start losing interest. In a study conducted by Kassebaum et al., students reported that preparing for written examinations was easier using the lecture method [18].

There were certain limitations to our study, as the number of faculty to facilitate CBL was less. More faculty involvement would have led to better student-teacher interaction. Due to a paucity of time, only four topics could be covered by CBL sessions. Also, since CBL was not included in university exams for assessment, some students were apprehensive about participating. To bring in some newer methodologies into our teaching and learning, sensitization and training of the faculty are must. Regular interaction among the teaching faculty would help clear some of the barriers.

## Conclusions

CBL was well received by the students and was thought to be viable and appropriate to be included in the curriculum, albeit requiring quite a bit of effort from them. It is essential that more contemporary interactive teaching strategies, like CBL, be incorporated into pharmacology instruction in light of the aforementioned findings and the rollout of the new National Medical Commission competency-based curriculum. The present study came to the conclusion that CBL is substantially more successful than traditional education at enhancing pharmacology understanding. A solid foundation will be created through the judicious and proper application of the CBL approach in conjunction with other conventional teaching techniques during the students' medical training, with the goal of producing an Indian medical graduate who is competent and self-assured and who is prepared to serve society. We have only looked at the results of CBL in the short term. Looking at the long-term effects, which may occur over the following years of clinical exposure and internship, is necessary to determine whether this knowledge results in better prescription abilities. It will complement the existing pharmacology teaching resources. To determine whether it can replace conventional teaching and learning methods, more research is required.
